# Efficiency of Hesperidin against Liver Fibrosis Induced by Bile Duct Ligation in Rats

**DOI:** 10.1155/2023/5444301

**Published:** 2023-04-11

**Authors:** Zahra Nasehi, Nejat Kheiripour, Maryam Akhavan Taheri, Abolfazl Ardjmand, Faezeh Jozi, Mohammad Esmaeil Shahaboddin

**Affiliations:** ^1^Department of Clinical Biochemistry, Faculty of Medicine, Kashan University of Medical Sciences, Kashan, Iran; ^2^Institute for Basic Sciences, Research Center for Biochemistry and Nutrition in Metabolic Diseases, Kashan University of Medical Sciences, Kashan, Iran; ^3^Institute for Basic Sciences, Anatomical Sciences Research Center, Kashan University of Medical Sciences, Kashan, Iran; ^4^Institute for Basic Sciences, Physiology Research Center, Kashan University of Medical Sciences, Kashan, Iran

## Abstract

**Introduction:**

Bile duct ligation (BDL) and subsequent cholestasis are associated with oxidative stress and liver injury and fibrosis. Hesperidin (3,5,7-trihydroxyflavanone 7-rhamnoglucoside) is a flavanone glycoside abundant in citrus fruits. It has positive effects on diabetic retinopathy, reduced platelet aggregation, and blood flow alterations and has the potential to reduce liver injury in oxidative stress. The aim of this study was to evaluate the hepatoprotective effects of hesperidin on BDL-induced liver injury in rats.

**Methods:**

A total of 48 adult male Wistar rats were equally allocated to six eight-rat groups, namely, a healthy group, a sham group, a BDL+Vehicle group (BDL plus treatment with distilled water), a BDL+VitC group (BDL plus treatment with vitamin C 4.25 *μ*g/kg), a BDL+Hesp100 group (BDL plus treatment with hesperidin 100 mg/kg/day), and a BDL+Hesp200 group (BDL plus treatment with hesperidin 200 mg/kg/day). Treatments were orally provided for 21 consecutive days. Finally, rats were sacrificed through heart blood sampling. Blood samples were centrifuged, and liver function, oxidative stress, and antioxidant parameters were assessed. Liver tissue was also assessed for oxidative stress, antioxidant, and histological parameters. The expression of inflammatory genes, namely, TGF*β*1, iNOS, Caspase-3, and *α*-SMA, was measured through polymerase chain reaction. *Findings*. Hesperidin supplementation was associated with significant decrease in the levels of liver enzymes, bilirubin, nitric oxide, malondialdehyde, protein carbonyl, and inflammatory gene expression; significant increase in the levels of total antioxidant capacity, glutathione, and superoxide dismutase and catalase enzyme activity; and significant improvement in the histological morphology and structure of the liver parenchyma.

**Conclusion:**

Hesperidin has significant positive effects on liver morphology and structure, inflammation, fibrosis, and oxidative stress in rats with BDL-induced cholestatic liver injury.

## 1. Introduction

Liver fibrosis (LF) is a major global health problem characterized by the accumulation of extracellular matrix due to exposure to reactive oxygen/nitrogen species (RONS) and subsequent oxidative stress [1]. Recent reports show that 844 million people in the world suffer from chronic liver disease and two million die each year due to its complications [2]. Chronic liver disease, due to secondary liver injury of any cause, leads to progressive LF, impairment of liver structure, vascular changes, and inappropriate reconstruction of liver tissue [3]. The severity of LF determines the severity of the adverse effects of the disease, including cirrhosis, hepatic cell carcinoma, and death [4].

Oxidative stress is a harmful process which can damage cellular structures. In chronic liver disease, such as LF, RONS play a significant role in the onset and progression of liver disease through activating several molecular pathways involved in the activation of the hepatic stellate cells and causing molecular changes in hepatocytes [5]. Moreover, inflammatory response after liver injury activates the different pathways of lymphocyte recruitment and migration which have direct relationship with the type of injury. Constant presence of LF stimuli can lead to cellular injury and fat accumulation and increase the risk of severe liver disease [6]. Therefore, antioxidants are considered as agents with potential positive effects on LF.

Hesperidin (3,5,7-trihydroxyflavanone 7-rhamnoglucoside), known as vitamin P, is an abundant and inexpensive flavanone glycoside extracted from citrus fruits such as lemon, orange, and grapefruit [7]. It is one of the safest and most important bioflavonoids with a wide range of known pharmacological properties [8] such as anti-inflammatory, antimicrobial, antioxidant, antineoplastic, antihypertensive, and immunity boosting effects [9]. Its anti-inflammatory effects are mainly due to its antioxidant effects and suppression of proinflammatory cytokines [10]. Hesperidin inhibits the production of proinflammatory cytokines such as interleukin 2 (IL-2) and interferon gamma (IFN-*γ*) [11] and inhibits inflammatory reactions stimulated by interleukin 1 beta (IL-1*β*) through inhibiting the activation of the nuclear factor kappa B (NF-*κ*B) signaling cascade [12]. Moreover, a study reported that hesperidin had significant role in the suppression of inflammatory markers such as tumor necrosis factor alpha (TNF*α*) and interleukin 6 (IL-6) in patients with type 2 diabetes mellitus [13]. In animal studies, hesperidin showed good safety profile with a lethal dose of 4837.5 mg/kg, while its long-term administration with a dose of 500 mg/kg caused no alteration in body weight, hematologic parameters, and clinical manifestations [9].

A similar study was conducted by Kong et al., which solely focuses on the short-term (1 week) anti-inflammatory effects of hesperidin in association with TGF-*β*1/Smad pathways on liver fibrosis in mice [14], whereas our study in addition to the anti-inflammatory properties examined oxidative and antioxidant pathways as well in rats. On the other hand, contrasting doses and 3-week treatment were selected in the current research. Hence, our study was conducted to fill the void left by Kong et al.'s study.

Despite its antioxidant effects and its potential protective effects against cholestatic liver injury, no study had yet evaluated the effects of hesperidin on inflammatory, oxidative, and antioxidant pathways on LF induced by bile duct ligation. Therefore, the present study was conducted to narrow this gap. The aim of the study was to evaluate the hepatoprotective effects of hesperidin on LF induced by bile duct ligation (BDL) in rats.

## 2. Methods

### 2.1. Materials

Ethylenediaminetetraacetic acid (EDTA), tetraethoxypropane (TEP), chloroform, N-(1-naphthyl)-ethylenediamine dihydrochloride (NEDD), sodium acetate, Triton X-100, vanadium chloride, ascorbic acid, 4-(2-hydroxyethyl)-1-piperazineethanesulfonic acid (HEPES), hydrogen peroxide, ethyl acetate, Coomassie blue, sodium nitrite, dithiothreitol (DTT), sulfanilamide, and Tris base were purchased from Merck (Darmstadt, Germany). Thiobarbituric acid (TBA), trichloroacetic acid (TCA), 2-amino-2-hydroxymethyl-propane-1,3-diol-hydrochloride (Tris-HCl), phenylmethylsulfonyl fluoride (PMSF), ammonium molybdate, diethyl pyrocarbonate (DEPC), p-dimethylaminobenzaldehyde, 2,4-dinitrophenylhydrazine (DNPH), bovine serum albumin (BSA), 5,5-dithionitrobenzoic acid (DTNB), metaphosphoric acid, guanidine hydrochloride, glacial acetic acid, boric acid, and isopropanol were purchased from Kalazist Co. (Tehran, Iran). 2,4,6-Tripyridyl-S-triazine (TPTZ) and hesperidin were purchased from Sigma-Aldrich (Missouri, USA). Formalin, hydrochloric acid (HCl), and ethanol were purchased from Mojallali Co. Tehran, Iran. Hesperidin was bought from the MilliporeSigma Company (St. Louis, MO, USA), kits for serum biochemical analysis were bought from the Pars Azmoon Company (Tehran, Iran), and kits for superoxide dismutase (SOD) assay were bought from the Kiazist Company (Hamadan, Iran). Other chemicals used in this study were research-grade materials.

### 2.2. Design

This experimental study was conducted in 2020 in the Clinical Biochemistry and the Physiology Research Center of Kashan University of Medical Sciences, Kashan, Iran.

### 2.3. Animals and Intervention

A total of 48 adult male Wistar rats (200–250 grams) were housed in a controlled animal center with a temperature of 25 ± 0.5°C, humidity of 50%–70%, 12 : 12 light-dark cycles, and free access to food and water. They were equally allocated to six eight-rat groups, namely, a healthy group, a sham group, a BDL+Vehicle group (BDL plus treatment with distilled water), a BDL+VitC group (BDL plus treatment with vitamin C 4.25 *μ*g/kg), a BDL+Hesp100 group (BDL plus treatment with hesperidin 100 mg/kg/day), and a BDL+Hesp200 group (BDL plus treatment with hesperidin 200 mg/kg/day). Hesperidin was dissolved in normal saline at two concentrations, including 100 and 200 mg/kg body weight, and administrated to experimental rats once daily for 21 consecutive days through gastric gavage. Hesperidin was freshly prepared every day and administrated at the same time throughout the treatment period [15]. The selection of two doses of hesperidin (100 and 200 mg/kg body weight) was made according to earlier conducted studies [16]. Likewise, L-ascorbic acid (4.25 g/mL) which is considered a low dose was selected as a positive reference antioxidant based on previous studies [17]. In addition, using the DPPH method, hesperidin extract showed higher potent scavenging activity of free radicals as compared to vitamin C [17]. For BDL under general anesthesia (intraperitoneal xylazine 10 mg/kg and ketamine 70 mg/kg), the common bile duct was accessed through an abdominal midline incision, ligated at two points, and cut between the two ligated points. Sham surgery included laparotomy to access the common bile duct without any manipulation of the duct. At the end of the three-week course of the intervention, heart blood samples were obtained under deep anesthesia and sample serum was separated through centrifuging at 3000 *g* for fifteen minutes and kept at a temperature of −20°C for later analysis. Immediately after sacrificing rats, liver tissue was separated and divided into three parts. One part was frozen in liquid nitrogen for ribonucleic acid (RNA) extraction, one part was kept at a temperature of −80°C to prepare tissue homogenate for the evaluation of antioxidant parameters, and one part was fixed using 10% formalin for histopathological assessments.

### 2.4. Biochemical Analysis

Standard kits and an automatic biochemistry analyzer (Biotecnica BT3000, Italy) were used to measure the serum levels of alanine aminotransferase (ALT), aspartate aminotransferase (AST), lactate dehydrogenase (LDH), alkaline phosphatase (ALP), and bilirubin.

### 2.5. Histopathological Assessment

The histopathological assessment of extrahepatic cholestatic structure was performed through eosin-hematoxylin staining. Micrograph images were obtained using a digital camera connected to an optical microscope (Nikon, Japan). In each slide, ten fields were randomly selected and scored using the METAVIR system. LF severity was scored as follows: 0: healthy liver; 1: collagen infiltration in some portal spaces without spread to the septum; 2: collagen infiltration to most portal spaces with spread to the septum and incomplete retraction of the portal duct to the central vein; 3: incomplete cirrhosis and complete connections among the septi so that the septi divide parenchyma into separate spaces; and 4: complete cirrhosis with complete and thick septum. The infiltration of lymphocyte inflammatory cells was assessed using the following scale: 0: no inflammation; 1: focal presence of inflammatory cells in less than 25% of liver tissue; 2: focal presence of inflammatory cells in 25%–50% of liver tissue; 3: focal presence of inflammatory cells throughout liver tissue; and 4: nonfocal presence of inflammatory cells throughout liver tissue. Bile duct hyperplasia was also measured using the following scale: 0: no duct hyperplasia; 1: hyperplasia in less than 25% of each lobule; 2: hyperplasia in 25%–50% of each lobule; 3: focal but extensive hyperplasia with nodule formation; and 4: diffuse hyperplasia and complete nodule formation. The scale for necrosis assessment was as follows: 0: no injury; 1: local injury in less than 25% of liver tissue; 2: local injury in 25%–50% of liver tissue; 3: local but extensive injury of liver tissue; and 4: extensive necrosis of hepatocytes [18].

### 2.6. Concentration of Total Nitrate

The levels of nitrite and nitrate, as indices of nitric oxide (NO) formation, were measured through the Griess reaction [19]. Using sodium nitrite as standard (0–50 *μ*mol/L), the levels of NO metabolites were reported as *μ*mol/L for serum and *μ*mol/mg protein for tissue.

### 2.7. Lipid Peroxidation Level

Protein level was measured according to the Bradford method (1976) using bovine serum albumin [20]. Lipid peroxidation level in liver homogenate was also measured through measuring malondialdehyde (MDA) formation using the thiobarbituric acid method [21, 22].

### 2.8. Protein Carbonyl (PC) Level

PC content was measured through spectrophotometric method based on the color produced during the reaction of 2,4-dinitrophenylhydrazine and carbonyl groups. PC level was determined through measuring absorption at 33 nm using a molar absorption coefficient of 2.2 × 10^4^ M^–1^cm^–1^, and the final results were reported as *μ*mol/L for serum and *μ*mol/mg protein for tissue [23].

### 2.9. Total Antioxidant Capacity (TAC)

The ferric reducing antioxidant power (FRAP) measurement was used to determine TAC based on serum and tissue ferric reducing ability which was estimated from the reduction of FE^3+^-TPTZ complex to FE^2+^ in low pH. FRAP content was expressed as mmol/L for plasma and mmol/mg protein for tissue using FeSO_4_·7H_2_O as standard (0.1–1 mmol/L) [24, 25].

### 2.10. Antioxidant Enzyme Activity and Glutathione (GSH) Levels

#### 2.10.1. SOD

Cytoplasmic and mitochondrial SOD activity was measured through spectrophotometric method. Radical reaction of SOD with resazurin leads to the formation of the resorufin compound which has a light absorption of 570 nm. SOD catalyzes the dismutation of superoxide radicals and thereby prevents their reaction with resazurin. SOD activity was determined through measuring its ability to prevent resazurin reduction, and the results were reported as U/mg protein.

#### 2.10.2. Catalase

Catalase activity was measured through the methods described by Hadwan and Abed, in which enzyme activity was determined through measuring absorption reduction in a medium with 50 mmol/L phosphate-buffered saline (pH: 7.4) and 20 mmol/L hydrogen peroxide. Enzyme activity was assessed via spectrophotometric method at a wavelength of 374 nm and was reported as kU/L for serum and kU/mg protein for tissue.

#### 2.10.3. GSH

The Ellman reagent 5,5′-dithiobis-(2-nitrobenzoic acid) (DTNB) was initially used to estimate thiol groups. This process is based on the reaction of DTNB with thiol for the production of the mixed 2-nitro-5-thiobenzoic acid (TNB) and disulfide complex which is determined with the TNB_2_ absorption at 412 nm. GSH content was calculated using the molar absorption coefficient of 1.36 × 10^3^ M^–1^cm^–1^ and was reported as mmol/L for plasma and mmol/mg protein for tissue [26].

### 2.11. Gene Expression

The levels of the expression of *β*-actin, transforming growth factor beta (TGF*β*1), inducible nitric oxide synthase (iNOS), Caspase-3, and alpha smooth muscle actin (*α*-SMA) were measured through quantitative polymerase chain reaction (qPCR) using the asymmetrical cyanine dye of SYBR Green on Bio-Rad MyiQ™ (Bio-Rad Laboratories, Inc., Hercules, CA). Complementary deoxyribonucleic acid (cDNA) synthesis was performed through the reverse transcription of total RNA (100 ng) using the Easy cDNA synthesis kit (Pars Tous Biotechnology, Iran). Forward and reverse primer sequences (5′→3′) were as follows: *β*-actin (forward): CTGTGTGGATTGGTGGCTCT; *β*-actin (reverse): CAGCTCAGTAACAGTCCGCC; TGF*β*-1 (forward): AGGGCTACCATGCCAACTTC; TGF*β*-1 (reverse): CCACGTAGTAGACGATGGGC; iNOS (forward): CCTCAGGCTTGGGTCTTGTTA; iNOS (reverse): CATCCTGTGTTGTTGGGCTG; Caspase-3 (forward): GGAGCTTGGAACGCGAAGAA; Caspase-3 (reverse): ACACAAGCCCATTTCAGGGT; *α*-SMA (forward): CAGCTATGTGGGGGACGAAG; *α*-SMA (reverse): TCCGTTAGCAAGGTCGGATG.

Relative gene expression was calculated as 2^–ΔΔCT^.

### 2.12. Statistical Analysis

Data were presented as mean ± standard deviation (mean ± SD) and analyzed using the one-way analysis of variance and Tukey's post hoc test at a significance level of less than 0.05. The SPSS software (Chicago, USA) was employed for data management and analysis.

### 2.13. Ethical Considerations

This study has the approval of the Ethics Committee of Kashan University of Medical Sciences, Kashan, Iran (code: IR.KAUMS.MEDNT.REC.1397.114). All methods of the study were in accordance with the ethical guidelines of this university in order to reduce rats' suffering.

## 3. Findings

### 3.1. Histopathological Assessment of Liver Tissue

**Figure 1:**
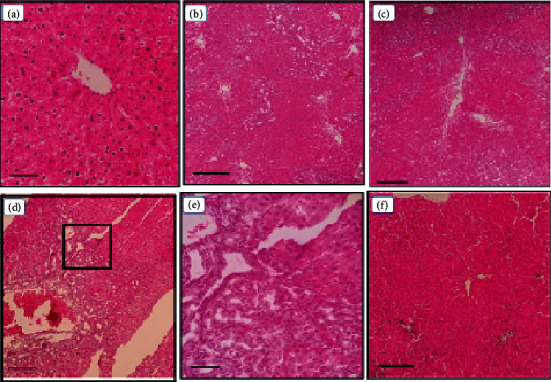
Effects of hesperidin on the histopathological changes of the liver in BDL-afflicted rats. Representative photomicrographs of liver sections processed for H and E staining (10; scale bar 5 mm). Sham ×400 (a); BDL-control ×100 (b); BDL+VitC ×100 (c); BDL+Hesp100 ×100 (d); BDL+Hesp100 ×400 (e); and BDL+Hesp200 ×100 (f). (a) represents the normal liver histopathology. (e) is a section of (d) at ×400 magnification. In (b)–(e), bile duct hyperplasia is evident. All these lesions markedly decreased in rats treated with 200 mg/kg hesperidin.

Liver tissues of all rats in the healthy and the sham groups had normal structure (score: 0 ± 0) with normal morphology of the liver parenchyma and healthy hepatocytes, sinusoids, and portal ducts (Figure 1). Histopathological assessment in the BDL+Vehicle group showed extensive collagen infiltration, fibrosis, and necrosis as well as inflammation and hyperplasia of the bile ducts. Rats in both the BDL+Hesp100 and BDL+Hesp200 groups had some levels of LF. However, the morphology and structure of the liver parenchyma in the BDL+Hesp200 group were significantly better than those in the BDL+Vehicle group, and liver tissue inflammation in the BDL+Hesp100 group was significantly less than that in the BDL+Vehicle group (*P* < 0.05). Moreover, liver morphology properties such as LF, inflammation, and necrosis as well as bile duct hyperplasia in both the BDL+Hesp100 and BDL+Hesp200 groups were significantly less than those in the BDL-VitC group (*P* < 0.05) (Table 1).

### 3.2. Biochemical Analysis

The serum levels of AST, ALT, ALP, LDH, and total and direct bilirubin in the BDL+Vehicle group were significantly more than those in the healthy and the sham groups (*P* < 0.05). It seems that abdominal incision, prodding of the bile duct, and hemolysis caused due to surgery may have resulted in the increase of liver enzymes in sham-operated rats as compared to healthy rats.

Moreover, the levels of these factors in the BDL+Hesp100 and BDL+Hesp200 groups were significantly less than those in the BDL+Vehicle group (*P* < 0.05) (Table 2), denoting that hesperidin reduces BDL-associated liver injury. Furthermore, the levels of ALT and total and direct bilirubin in both the BDL+Hesp100 and BDL+Hesp200 groups and the levels of AST, ALP, and LDH in the BDL+Hesp200 group were significantly less than those in the BDL+VitC group (*P* < 0.05) (Table 2).

### 3.3. Levels of NO in Serum and Liver Tissue

**Figure 2:**
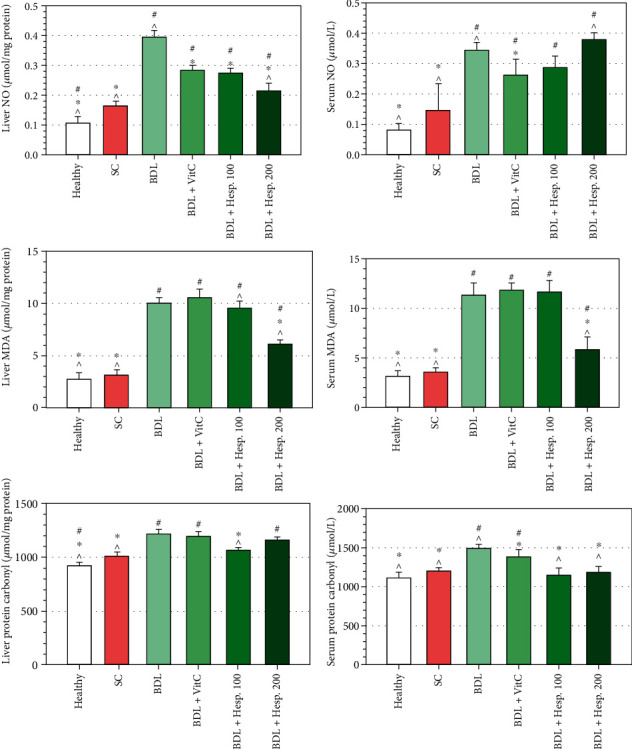
The levels of nitric oxide, malondialdehyde (MDA), and protein carbonyl in liver tissue and serum in different groups. Data are presented as mean ± SD. SC: sham control; BDL: bile duct ligation; VitC: vitamin C; Hesp: hesperidin. ^#^Significant difference with the sham group. ^∗^Significant difference with the BDL+Vehicle group. ^^^Significant difference with the BDL+VitC group.

The level of NO in liver tissue in the BDL+Vehicle group was significantly greater than that in the sham group (*P* < 0.05), and the levels of NO metabolites in liver tissue in both the BDL+Hesp100 and BDL+Hesp200 groups were significantly less than those in the BDL+Vehicle group (*P* < 0.05). Moreover, the level of NO in liver tissue in the BDL+Hesp200 group was significantly less than that in the BDL+VitC group (*P* < 0.05), while there was no significant difference between the BDL+Hesp100 and the BDL+VitC groups respecting the levels of NO in liver tissue (*P* > 0.05) (Figure 2). However, the level of serum NO in the BDL+Hesp100 and BDL+Hesp200 groups did not significantly differ from that in the BDL+Vehicle group (*P* > 0.05).

### 3.4. MDA Level in Serum and Liver Tissue

MDA level in liver tissue in the BDL+Vehicle group was significantly more than that in the sham group (*P* < 0.05). Moreover, MDA level in liver tissue of the BDL+Hesp200 group was significantly less than that of the BDL+Vehicle group (*P* < 005), while there was no significant difference between the BDL+Vehicle and BDL+Hesp100 groups respecting MDA level (*P* > 0.05). MDA level in liver tissue in both the BDL+Hesp100 and BDL+Hesp200 groups was significantly less than that in the BDL+VitC group (*P* < 0.05) (Figure 2). Moreover, serum MDA level in both the BDL+Hesp100 and BDL+Hesp200 groups was significantly less than that in the BDL+Vehicle group (*P* < 0.05) (Figure 2). Furthermore, serum MDA level in the BDL+Hesp200 group was significantly less than that in the BDL+VitC group (*P* < 0.05), while the difference between the BDL+Hesp100 and BDL+VitC groups was not significant (*P* > 0.05; Figure 2).

### 3.5. PC Level in Serum and Liver Tissue

PC level in liver tissue in the BDL+Vehicle group was significantly more than that in the sham group. Moreover, PC level in liver tissue in the BDL+Hesp100 group was significantly less than that in the BDL+Vehicle group (*P* < 0.05), while the difference between the BDL+Hesp200 and BDL+Vehicle groups was not significant (*P* > 0.05). Serum PC level in the BDL+Hesp200 group was significantly less than that in the BDL+Vehicle group (*P* < 0.05).

### 3.6. TAC Level in Serum and Liver Tissue

**Figure 3:**
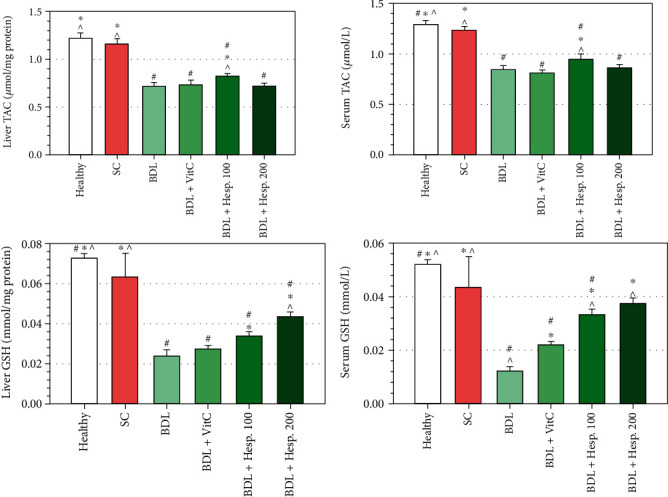
The levels of total antioxidant capacity (TAC) and glutathione (GSH) in liver tissue and serum in different groups. Data are presented as mean ± SD. SC: sham control; BDL: bile duct ligation; VitC: vitamin C; Hesp: hesperidin. ^#^Significant difference with the sham group. ^∗^Significant difference with the BDL+Vehicle group. ^^^Significant difference with the BDL+VitC group.

TAC level in liver tissue in the BDL+Vehicle group was significantly less than that in the sham group. Moreover, TAC level in serum and liver tissue in the BDL+Hesp100 group was significantly more than that in the BDL+Vehicle group (*P* < 0.05), but the difference between the BDL+Hesp200 and BDL+Vehicle groups was not significant (*P* > 0.05) (Figure 3). Furthermore, there was no significant difference between the BDL+Hesp200 and BDL+VitC groups respecting TAC level in serum and liver tissue (*P* > 0.05), while TAC level in serum and liver tissue in the BDL+Hesp100 group was significantly greater than that in the BDL+VitC group (*P* < 0.05). Interestingly, the BDL+Hesp100 group displayed higher levels of TAC as compared to the BDL+Hesp200 group (Figure 3).

### 3.7. Thiol Group Level in Serum and Liver Tissue

GSH level in liver tissue in the BDL+Vehicle group was significantly less than that in the sham group (*P* < 0.05), and GSH level in liver tissue in both the BDL+Hesp100 and BDL+Hesp200 groups was significantly more than that in the BDL+Vehicle group (*P* < 0.05) (Figure 3). Moreover, GSH level in liver tissue in the BDL+Hesp200 group was significantly more than that in the BDL+VitC group (*P* < 0.05) (Figure 3). On the other hand, serum GSH level in both the BDL+Hesp100 and BDL+Hesp200 groups was significantly more than that in the BDL+Vehicle group (*P* < 0.05).

### 3.8. SOD and Catalase Enzyme Activity

**Figure 4:**
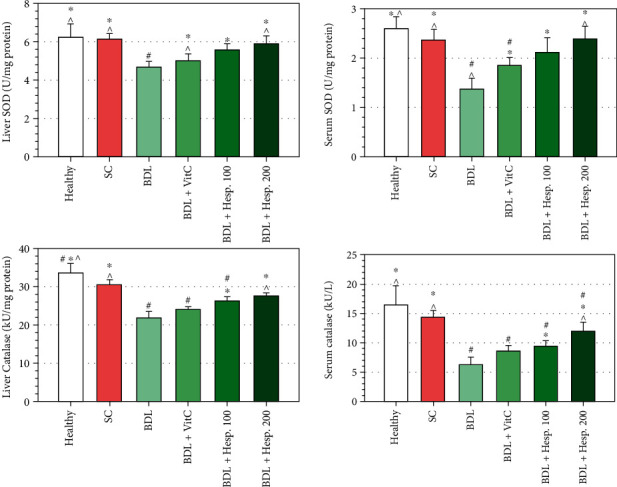
The levels of superoxide dismutase (SOD) and catalase enzyme activity in liver tissue and serum in different groups. Data are presented as mean ± SD. SC: sham control; BDL: bile duct ligation; VitC: vitamin C; Hesp: hesperidin. ^#^Significant difference with the sham group. ^∗^Significant difference with the BDL+Vehicle group. ^^^Significant difference with the BDL+VitC group.

SOD enzyme activity in liver tissue in the BDL+Vehicle group was significantly less than that in the sham group (*P* < 0.05) (Figure 4). SOD and catalase enzyme activity in liver tissue in both the BDL+Hesp100 and BDL+Hesp200 groups was significantly more than that in the BDL+Vehicle group (Figure 4). SOD and catalase enzyme activity in the BDL+Hesp200 group was significantly more than that in the BDL+VitC group (*P* < 0.05), while the difference between the BDL+Hesp100 and BDL+VitC groups was not significant (*P* > 0.05) (Figure 4). SOD and catalase enzyme activity in serum in both the BDL+Hesp100 and BDL+Hesp200 groups was significantly more than that in the BDL+Vehicle group (*P* < 0.05) (Figure 4). Moreover, SOD and catalase enzyme activity in serum in the BDL+Hesp200 group was significantly more than that in the BDL+VitC group (*P* < 0.05).

### 3.9. Gene Expression

**Figure 5:**
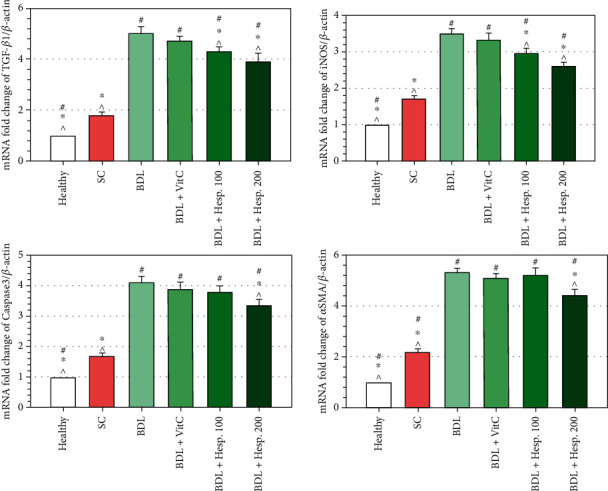
The level of the *β*-actin, TGF*β*1, iNOS, Caspase-3, and *α*-SMA gene expression in different groups. Data are presented as mean ± SD. SC: sham control; BDL: bile duct ligation; VitC: vitamin C; Hesp: hesperidin; TGF*β*1: transforming growth factor *β*1; iNOS: inducible nitric oxide synthase; *α*-SMA: alpha smooth muscle actin. ^#^Significant difference with the sham group. ^∗^Significant difference with the BDL+Vehicle group. ^^^Significant difference with the BDL+VitC group.

The results of real-time PCR revealed the augmented expression of the inflammatory genes so that the level of TGF*β*1, iNOS, Caspase-3, and *α*-SMA gene expression in liver tissue in the BDL+Vehicle group was significantly more than that in the sham group by five, 3.5, 4.1, and 5.4 times, respectively (*P* < 0.05) (Figure 4). On the other hand, the level of TGF*β*1 and iNOS gene expression in liver tissue in both the BDL+Hesp100 and BDL+Hesp200 groups was significantly less than that in the BDL+Vehicle group (Figure 4), while the level of Caspase-3 and *α*-SMA gene expression was significantly less than that in the BDL+Vehicle group just in the BDL+Hesp200 group (*P* < 0.05) (Figure 5). Moreover, the level of the TGF*β*1 and iNOS gene expression in both the BDL+Hesp100 and BDL+Hesp200 groups was significantly less than that in the BDL-VitC group (*P* < 0.05), and the level of Caspase-3 and *α*-SMA gene expression in the BDL+Hesp200 group was significantly less than that in the BDL-VitC group (*P* < 0.05).

### 3.10. Mortality

The mortality rate during the 21 days of treatment was 25% (12 rats). In the BDL-operated group (*n* = 12), 4 rats died (33.3%); in the BDL+Hesp100 group (*n* = 12), 3 rats died (25%); in the BDL+Hesp200 group (*n* = 12), 2 rats died (16.6%); and in the BDL+VitC group (*n* = 12), only 3 rats died (25%), without any significant difference between groups at the end of 21 days prior to fibrosis induction.

## 4. Discussion

This study evaluated the hepatoprotective effects of hesperidin on BDL-induced LF in rats. The findings revealed that hesperidin supplementation had significant protective effects against cholestatic liver injury as confirmed by positive changes in histopathological, serum biochemical, fibrotic, inflammatory, and oxidative stress parameters.

Study findings showed that the level of NO metabolites in liver tissue in the BDL+Vehicle group was significantly more than that in the sham, BDL+Hesp100, and BDL+Hesp200 groups. This is in line with the findings of a previous study [27]. NO is a highly reactive molecule produced in hepatocytes. Hesperidin inhibits NO production through suppressing the expression of the iNOS enzyme [28]. Reduction of reactive nitrogen species such as NO at intracellular level through suppressing the iNOS enzyme expression seems to reduce oxidative stress and prevent further liver injury. Our findings also showed that despite its significant positive effects on NO in liver tissue, hesperidin had no significant effects on serum NO level. This finding may be due to the high volatility of NO. A study showed that measurement of tissue NO provides more reliable results than serum NO measurement [19].

We also found that MDA level in the BDL+Vehicle group was significantly more than that in the sham group. A previous study also showed that BDL-induced cholestatic liver injury in rats was associated with significant increase in MDA level [29]. Our findings showed that treatment with hesperidin 200 mg/kg/day for 21 days significantly reduced tissue and serum MDA level, while treatment with hesperidin with a dose of 100 mg/kg/day had no significant effects on MDA level. MDA is a tissue injury marker released from the liver of rats afflicted by BDL-induced LF due to the toxicity of reactive oxygen species. In agreement with our findings, previous studies reported the protective effects of some antioxidants such as curcumin against BDL-associated liver injury [30, 31]. Another study reported that hesperidin intake for six weeks significantly reduced MDA level among patients with type 2 diabetes mellitus [32]. Decrease in MDA level after hesperidin intake denotes the antioxidant potentials of this flavonoid. It is noteworthy that hesperidin exerts its hepatoprotective effects through reducing lipid peroxidation [33].

Study findings also showed that PC level serum and liver tissue in the BDL+Vehicle group was significantly greater than that in the sham group and significantly less than that in the BDL+Hesp100 and the BDL+Hesp200 groups. In agreement with these findings, two previous studies reported oxidative injury to proteins and liver in cholestatic animal models such as CCL_4_ and BDL [34, 35]. Moreover, a study showed that hesperidin therapy suppressed hyperglycemia-induced PC production in the retinal ganglion cells [36]. Although the products of oxidative stress react to lipids, proteins, or DNA, proteins which often act as enzyme in cells are more vulnerable to these products. PC is a principal marker of protein oxidation due to free radicals [37]. Therefore, hesperidin can suppress protein and lipid peroxidation and increase antioxidant capacity in rats with BDL.

We also found that TAC and GSH levels in the BDL+Vehicle group were significantly less than those in the sham and BDL+Hesp100 groups. Similarly, a study showed that herbs with antioxidant compounds, such as watercress, increased TAC level by 8% in rats with BDL [38]. GSH and FRAP indicate the antioxidant capacity of biological fluids or tissues. Increase in TAC level in rats treated with hesperidin in the present study can reflect the antioxidant effects of hesperidin. However, a study reported the higher level of FRAP in rats with BDL compared with rats in the sham groups [27]. This contradiction may be due to the high level of bilirubin as a potent antioxidant in that study.

We also found that SOD and catalase enzyme activity in the BDL+Vehicle group was significantly less than that in the sham group. Antioxidant enzymes such as SOD and catalase can protect cellular structures against damages caused by reactive oxygen species. Two previous studies showed that the antioxidant activity of these enzymes reduced seven days after BDL [39, 40], while a study reported no significant change in SOD and catalase enzyme activity ten days after BDL [41]. This contradiction is attributable to the differences among studies respecting their animal models and intervention duration. Our findings also showed that SOD and catalase enzyme activity in both the BDL+Hesp100 and BDL+Hesp200 groups was significantly more than that in the BDL+Vehicle group. This is in agreement with the findings of a previous study which reported that hesperidin significantly improved SOD and catalase enzyme activity in rats with myocardial ischemia [42]. These findings support this hypothesis that as potent free radical scavenger, hesperidin effectively controls the levels of reactive oxygen species during BDL-induced stress through regulating both enzymatic and nonenzymatic antioxidant defense systems.

Study findings also showed the higher level of the TGF*β*1 gene expression in the BDL+Vehicle group compared with the sham group. Similarly, a study reported that BDL significantly increased profibrogenic cytokines such as TGF*β* and extracellular matrix components such as *α*-SMA in hepatic proteins and gene expression which significantly decreased after treatment with ethyl acetate fraction of Amomum xanthioides [43]. LF pathogenesis includes multiple mechanisms such as inflammatory pathways, growth factor signaling, and lipid signaling. TGF*β*-mediated inflammatory pathways and growth factor signaling are the most important pathways of LF. The suppression of TGF*β*1 activity in animal models using antioxidants such as hesperidin can be an effective method to inhibit fibrotic response to hepatic injury.

Another finding of the present study was that compared with the sham group and both the BDL+Hesp100 and BDL+Hesp200 groups, iNOS gene expression was higher in the BDL+Vehicle group. A study showed that NF-*κ*B activation led to the upregulation of the replication of several genes involved in inflammatory pathways and apoptosis such as iNOS [44]. During oxidative stress, reactive oxygen species can activate gene expression through NF-*κ*B paths and thereby upregulate different genes such as iNOS [45]. Increased iNOS gene expression and NO production start the apoptosis cascade which can alter the normal functioning of tissues and lead to inflammation, apoptosis, and other complications in the liver. This path might have contributed to the development of LF in the present study, while hesperidin might have reduced iNOS expression, NO production, inflammation, and LF [44].

In the present study, the Caspase-3 gene expression in the BDL+Hesp100 group was significantly less than that in the BDL+Vehicle group. A study found high levels of Caspase-3 in the CCl_4_-induced LF group compared with the control group which significantly decreased after treatment with salvianolic acid A. Apoptosis is a proinflammatory process with a significant role in LF. Caspase-3 is the main enzyme in this process which leads to the enzymatic events that cause cell death [46].

We also found that the protective effects of hesperidin supplementation at both 100 and 200 mg/kg/day doses were significantly more than supplementation with vitamin C at a dose of 4.25 *μ*g/kg. Another study into the TAC of hesperidin and vitamin C also reported the greater antioxidant effects of hesperidin compared with vitamin C [47].

The findings also showed that hesperidin 200 mg/kg/day was more effective than hesperidin 100 mg/kg/day in reducing cholestatic liver injury. Assessment of the morphology and structure of the liver parenchyma also confirmed the greater positive effects of the hesperidin 200 mg/kg/day. Similarly, a study into the 25, 50, and 100 mg/kg/day doses of hesperidin reported its positive dose-dependent effects on blood glucose and insulin levels in rats with diabetes mellitus [48]. The antioxidant effects of hesperidin are attributable to its chemical structure, the arrangement of the hydroxyl group and the double bond, mutual configuration of the double bond and the carbonyl group of the C ring, and hydroxyl and methoxy system [47].

## 5. Conclusion

This study suggests that hesperidin can act as a hepatoprotective agent and exert more hepatoprotective effects than vitamin C. The hepatoprotective effects of hesperidin can be attributed to its free radical scavenging and antioxidant effects. Therefore, hesperidin can be used as a useful adjacent therapy to reduce cholestatic hepatic injuries.

## Figures and Tables

**Table 1:** Group comparisons respecting the characteristics of BDL-induced liver injury.

Group	Parameters			
	Fibrosis	Inflammation	Bile duct hyperplasia	Necrosis
BDL	3 ± 0.71^#^	2.6 ± 0.55^#^^	3 ± 0.71^#^	3.4 ± 0.55^#^
BDL+VitC (4.25 *μ*g/mL)	2 ± 0.71^#^	3.6 ± 0.55^#^^∗^	3.4 ± 0.89^#^	3.2 ± 0.84^#^
BDL+Hesp (100 mg/kg)	2.2 ± 0.84^#^	1.4 ± 0.55^#^^∗^^^^	3 ± 1^#^	2.4 ± 0.89^#^
BDL+Hesp (200 mg/kg)	1.8 ± 0.84^#^^∗^	1 ± 0^#^^∗^^^^	1.6 ± 0.55^#^^∗^^^^	2.2 ± 0.84^#^^∗^

Data are presented as mean ± SD. BDL: bile duct ligation; VitC: vitamin C; Hesp: hesperidin. ^#^Significant difference with the sham group. ^∗^Significant difference with the BDL+Vehicle group. ^^^Significant difference with the BDL+VitC group.

**Table 2:** Group comparisons respecting liver enzymes and serum bilirubin.

Group	Parameter					
	AST (IU/L)	ALT (IU/L)	ALP (IU/L)	LDH (IU/L)	Total bilirubin (mg/dL)	Direct bilirubin (mg/dL)
Healthy	182.8 ± 9.26^∗^^^^	81.6 ± 7.47^∗^^^^	544.6 ± 36.91^∗^^^^	2327.8 ± 141.81^∗^^^^	0.3 ± 0^∗^^^^	0.1 ± 0^∗^^^^
Sham	203.8 ± 15.87^∗^^^^	94.2 ± 7.01^∗^^^^	715.8 ± 69.32^∗^^^^	2621.2 ± 88.72^∗^^^^	0.4 ± 0^∗^^^^	0.1 ± 0^∗^^^^
BDL	268.4 ± 11.01^#^	150.2 ± 8.12^#^^	1316.2 ± 215.11^#^^	4084.8 ± 129.45^#^	8.08 ± 1.15^#^	6.1 ± 1.14^#^
BDL+VitC (4.25 *μ*g/kg)	249 ± 13.67^#^	108.6 ± 7.83^#^^∗^	1076.4 ± 117.43^#^^∗^	3784.2 ± 108.36^#^	6.44 ± 2.37^#^	5.26 ± 0.36^#^
BDL+Hesp (100 mg/kg)	230.2 ± 8.31^#^^∗^	134.4 ± 5.13^#^^∗^^^^	1069.6 ± 92.63^#^^∗^	3511 ± 196.19^#^^∗^	4.13 ± 0.33^#^^∗^^^^	2.28 ± 0.79^#^^∗^^^^
BDL+Hesp (200 mg/kg)	208.6 ± 10.5^∗^^^^	126.6 ± 6.43^#^^∗^^^^	786.6 ± 119.94^∗^^^^	3358 ± 132.78^#^^∗^^^^	2.77 ± 0.38^#^^∗^^^^	1.55 ± 0.28^#^^∗^^^^

Data are presented as mean ± SD (*n* = 8). BDL: bile duct ligation; VitC: vitamin C; Hesp: hesperidin; ALT: alanine aminotransferase; AST: aspartate aminotransferase; LDH: lactate dehydrogenase; ALP: alkaline phosphatase. ^#^Significant difference with the sham group. ^∗^Significant difference with the BDL+Vehicle group. ^^^Significant difference with the BDL+VitC group.

## Data Availability

Data are available on request.
